# The safety and efficacy of laparoscopic gastrectomy for patients with locally advanced gastric cancer following neoadjuvant chemotherapy

**DOI:** 10.1038/s41598-022-14717-6

**Published:** 2022-06-20

**Authors:** Lihang Liu, Chuandong Wang, Feng Li, Xiaojuan Zhang, Xuefei Cheng, Shengtao Lin, Yi Liu, Changshun Yang, Weihua Li

**Affiliations:** 1 Shengli Clinical Medical College, Fujian Medical University, No. 134, East Street, Fuzhou, 350001 China; 2 Department of Surgical Oncology, Fujian Provincial Hospital, Fuzhou, 350001 China; 3 Department of Pathology, Fujian Provincial Hospital, Fuzhou, 350001 China; 4 Fuzong Clinical Medical College, Fujian Medical University, Fuzhou, 350001 China; 5 Department of Endoscopy, National Cancer Center/Cancer Hospital, Chinese Academy of Medical Science, Peking Union Medical College, Beijing, People’s Republic of China

**Keywords:** Diseases, Oncology

## Abstract

Limited researches focused on the application of laparoscopic gastrectomy (LG) in locally advanced gastric cancer (LAGC) patients following neoadjuvant chemotherapy (NACT). In this study, we aimed at illustrating the surgical and survival outcome of LG in LAGC patients following NACT. We performed a retrospective study of patients with LAGC who received either LG following NACT or upfront LG at Fujian Provincial Hospital between March 2013 and October 2018. Perioperative parameters, short-term and long-term outcomes were compared. The Kaplan–Meier estimator was used to describe the survival curves, and the differences were examined by the log-rank test. In total, 76 consecutive patients were enrolled into the NACT-LG (41 patients) and LG (35 patients) group. The postoperative hospital stay was significantly longer for LG than for NACT-LG (11.0 vs. 12.0 day, P = 0.031). Significant difference was found in Grade ≥ III severe postoperative complications in two groups (0 vs. 17.1%, P = 0.001). No patient died of postoperative complications in the NACT-LG group, and one patient (1/35, 2.9%) died of postoperative complications in the LG group. A forest plot revealed that most subgroups of LG group were at great risks of postoperative complications. Compared with the LG group, the NACT-LG group had a significantly better DFS (14.4% vs. 5.7%, P = 0.0299) and better OS (34.1% vs. 8.6%, P = 0.0061) at 3 years. NACT increased the safety of LG for patients with LAGC and offer better disease-free and overall survival. For patients with LAGC, LG following NACT should be the priority treatment.

## Introduction

Gastric cancer is a life-threatening disease, and surgical resection remains the only curative treatment^1,2^. However, the long-term outcome is still far from satisfactory for patients who receive surgery alone, especially in patients with locally advanced gastric cancer (LAGC). Approximately 70% patients with LAGC died within 5 years after surgery^3,4^. Therefore, perioperative therapy is imperatively required to improve the survival.

Neoadjuvant chemotherapy (NACT) is generally accepted to benefit prognosis by downstaging tumor, increasing complete resection rate and eradicating micro-metastases^5,6^. The MAGIC study has illustrated a survival benefit of perioperative chemotherapy and consequently opened the era of neoadjuvant therapy in patients with LAGC^7^. Although NACT has demonstrated several strengths as mentioned above, surgeons concerned about its negative effects on the surgical safety. Destruction of anatomical dissection plane induced by tissue edema and fibrotic changes may complicate laparoscopic surgeries. In addition, chemotherapy related adverse effects deteriorate the nutritional and immune status of patients, which may impair the prognosis of patients^8–10^.

With the development of laparoscopic technique and accumulation of evidence from clinical trials in recent years, laparoscopic surgery has been recommended to patients with LAGC^11^. Nevertheless, limited studies focused on the application of laparoscopic gastrectomy (LG) in patients receiving NACT. There is an urgent need for researches comparing patients receiving laparoscopic surgery after NACT or upfront laparoscopic surgery. However, conducting prospective trials targeting on the issue is impractical for the reason that NACT has been widely accepted in patients with locally advanced gastric cancer (LAGC) due to its positive effects on patients’ survival^12–14^.

Therefore, this retrospective study was conducted to evaluate the safety and efficacy of LG for patients with LAGC following NACT, focusing on whether NACT increased the safety of LG for patients with LAGC.

## Patients and methods

### Patients

We reviewed our prospectively maintained gastric cancer database at Fujian Provincial Hospital. Clinical data of LAGC patients underwent either LG following NACT or upfront LG between March 2013 and October 2018 were analyzed. Patients were randomly assigned to the 2 groups. The inclusion criteria were listed as follows: (1) stomach adenocarcinoma, histologically confirmed by endoscopic biopsy; (2) clinical stage III (cT3/4a, N+, M0) according to the 8th edition of the AJCC/UICC staging system^15^, diagnosed using computed tomography (CT), endoscopic ultrasonography (EUS), or laparoscopic exploration; (3) totally laparoscopic gastrectomy. The exclusion criteria were listed as follows: (1) cancer of the esophagogastric junction; (2) residual gastric cancer; (3) malignant tumor history; and (4) emergency surgery due to complications (obstruction, bleeding, or perforation); (5) laparoscopy-assisted gastrectomy; (6) incomplete clinical and pathological data.

All patients signed written informed consent, and this study was approved by the ethics committee of the Fujian Provincial Hospital. The study was conducted in accordance with the ethical principles outlined in the Declaration of Helsinki, the national legislation, and the institutional requirements.

### Treatment

Patients in the NACT-LG group received three to six cycles of NACT (XELOX regimen) at three-weekly intervals. XELOX regimen consisted of oral fluoropyrimidine capecitabine (825 mg/m^2^, twice daily on days 1–14) and intravenous oxaliplatin (130 mg/m^2^ on day 1). Surgery was performed four to five weeks after the last cycle. Patients in the LG group received upfront surgery after preoperative evaluation. Laparoscopic total or distal gastrectomy with D2 lymphadenectomy was performed for patients according to the location of tumor. Reconstruction of the gastrointestinal tract was all completed using Roux-en-Y anastomosis. All the operations were performed by one experienced surgeon team. Within five weeks after surgery, patients were treated with three to six cycles of postoperative chemotherapy (XELOX regimen) at three-weekly intervals.

### Clinical and pathologic assessment

Clinicopathological variables, such as age, sex, body mass index (BMI), Eastern Clinical Oncology Group performance status (ECOG PS)^16^, tumor size, tumor location, Borrmann type, Lauren type, cT stage, as well as perioperative data, such as surgical type, incision length, operation time, blood loss, first aerofluxus time, drainage duration, postoperative hospital stay, and surgical radicalness were analyzed. Clavien-Dindo classification system was applied to evaluate postoperative complication, which occurs within 30 days^17,18^. Postoperative mortality was defined as death of any cause within 30 days after surgery. Pathological findings including number of harvested and metastatic lymph nodes, pathological stage, and tumor regression grade (TRG) were also compared between groups. The TRG was evaluated according to the NCCN guideline^12^.

### Follow-up

All the patients received postoperative follow-up every 3 months within 2 years after surgery and every 6 months for the next 3 years. Recurrence was defined as local recurrence identified by contrast CT scan or endoscopy biopsy, or distant recurrence identified by CT scan, ECT bone scan or PET/CT. Immediate follow-up occurred in patients with worsening or new symptoms. The disease-free survival (DFS) was calculated from surgery to recurrence, death, or last follow-up; the overall survival (OS) was calculated from beginning of treatment to death or last follow-up.

### Statistical analysis

Continuous variables with normal distribution were expressed as the mean ± standard deviation (SD) while non-normal variables were expressed as the median (range). T-test or Mann–Whitney rank-sum test was used to evaluate differences between the two groups according to the distribution of data. The Kaplan–Meier method was performed using a log-rank test to estimate differences in DFS and OS. SPSS version 20 was used for the statistical analysis, and P values less than 0.05 (two-sided) were considered statistically significant.

## Results

### Patients

**Figure 1:**
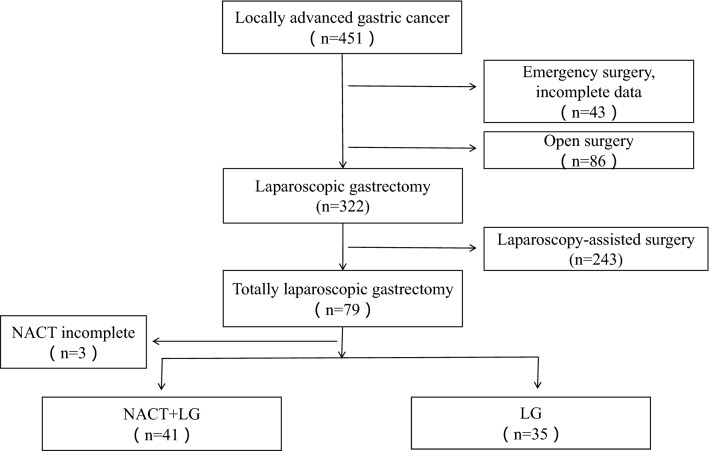
A flowchart presenting the selection procedure.

During the study period, we identified 451 patients with locally advanced gastric cancer. 375 patients were excluded due to emergency surgery and incomplete data (n = 43), open surgery (n = 86), laparoscopy-assisted surgery (n = 243), NACT incomplete which results from intolerable responses to chemotherapies (n = 3). We obtained data on76 patients in this study, 41 (53.9%) patients in the NACT-LG group and 35 (46.1%) patients in the LG group, respectively (Fig. 1). The characteristics of patients were detailed in Table 1. The two groups were balanced in baseline characteristics in term of age, sex, BMI and ECOG PS (all P > 0.05). There were also no differences between groups in tumor size, tumor location, Borrmann type, Lauren type, differentiation, cT stage and postoperative chemotherapy cycles (all P > 0.05).

**Table 1:** Patient baseline characteristics in the NACT-LG and LG groups.

	NACT-LG (n = 41)	LG (n = 35)	P
**Age (years)**			0.112
< 60	19 (46.3)	10 (28.6)	
≥ 60	22 (53.7)	25 (71.4)	
**Sex**			0.231
Male	32 (78.0)	23 (65.7)	
Female	9 (22.0)	12 (34.3)	
BMI (kg/m^2^)	23.2 ± 1.9	22.6 ± 2.8	0.238
**ECOG PS**			0.760
0	26 (63.4)	21 (60.0)	
1	15 (36.6)	14 (40.0)	
**Tumor size (cm)**			0.193
< 5	26 (63.4)	17 (48.6)	
≥ 5	15 (36.6)	18 (51.4)	
**Tumor location**			0.187
Upper third	15 (36.6)	6 (17.1)	
Middle third	14 (34.1)	14 (40.0)	
Lower third	12 (29.3)	14 (40.0)	
Total	0 (0)	1 (2.9)	
**Borrmann type**			0.117
I	1 (2.4)	2 (5.7)	
II	7 (17.2)	1 (2.9)	
III	32 (78.0)	29 (82.9)	
IV	1 (2.4)	3 (8.6)	
**Lauren type**			0.913
Diffused	9 (22.0)	9 (25.7)	
Intestinal	18 (43.9)	14 (40.0)	
Mixed	14 (34.1)	12 (34.3)	
**cT stage**			0.367
T3	18 (43.9)	19 (54.3)	
T4	23 (56.1)	16 (45.7)	
**Chemotherapy cycles**			
Preoperative	4 (3–6)	NA	NA
Postoperative	3 (3–6)	4 (3–6)	0.160

Fisher’s exact test was used as an alternative to Chi-square test when the number in one of the cells is smaller than 5.

*NACT* neoadjuvant chemotherapy, *LG* laparascopic gastrectomy, *BMI* body mass index, *ECOG PS* Eastern Clinical Oncology Group performance status, *NA* not applicable.

### Surgical procedures

All patients received an LG with D2 lymphadenectomy. The surgical data were compared in Table 2. No significant difference was found in the surgical trauma in terms of incision length operation time, blood loss, and postoperative recovery in terms of first aerofluxus time, and first time on liquid diets between the two groups (all P > 0.05). Patients in LG group had longer postoperative hospital stay (11.0 *vs.* 12.0d, P = 0.031). In addition, the NACT-LG exhibited a significantly greater R0 resection rate (95.1% *vs.* 77.1%, P < 0.05). R1 resection was performed in two (4.9%) patients in the NACT-LG group with positive proximal margins. Eight (22.9%) patients with R1 resection were found in the LG group, including four (11.4%) patients with intramural extension to esophagus and four (11.4%) patients with intramural extension to duodenum.. No similar situations were found in the NACT-LG group.

**Table 2:** Comparison of surgical procedures between the NACT-LG and LG groups.

	NACT-LG (n = 41)	LG (n = 35)	P
**Surgical type**			0.327
LTG	30 (73.2)	21 (60.0)	
LDG	11 (26.8)	14 (40.0)	
Incision length (cm)	6.0 (3.0–8.0)	5.0 (4.0–7.0)	0.109
Operation time (min)	260.0 (205.0–346)	250.0(140.0–360.0)	0.281
Estimated blood loss (mL)	100.0 (20.0–450.0)	100.0 (30.0–2500.0)	0.395
The first aerofluxus time (days)	3.0 (1.0–6.0)	3.0 (1.0–6.0)	0.303
Time to pull drainage (days)	8.0 (5.0–13.0)	7.0 (5.0–14.0)	0.061
First time on liquid diets (days)	3.0 (2.0–12.0)	3.0 (1.0–12.0)	0.118
Hospital stay after surgery (days)	11.0 (6.0–18.0)	12.0 (6.0–77.0)	0.031
**Surgical radicalness**			0.049
R0	39 (95.1)	27 (77.1)	
R1	2 (4.9)	8 (22.9)	

Fisher’s exact test was used as an alternative to Chi-square test when the number in one of the cells is smaller than 5.

*NACT* neoadjuvant chemotherapy, *LG* laparascopic gastrectomy, *LTG* laparoscopic total gastrectomy, *LDG* laparoscopic distal gastrectomy.

### Surgery morbidity and mortality

**Figure 2:**
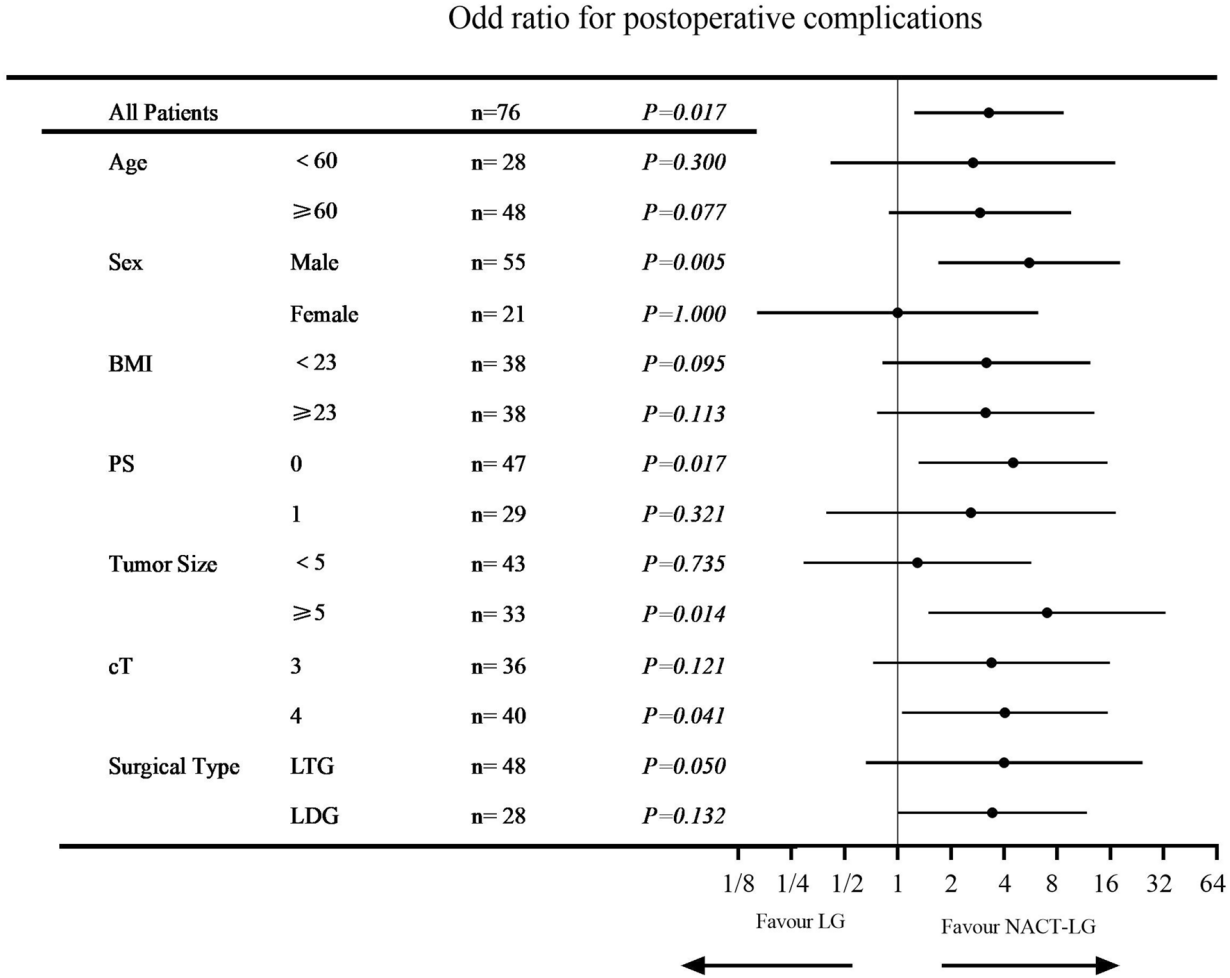
Forest plot evaluating the impact of the treatment selections on postoperative complications.

The postoperative complications were detailed in Table 3. Grade II postoperative complications occurred similarly in two groups, 9 patients (22.0%) in the NACT-LG group and 10 patients (28.5%) in the LG group (P = 0.680). The most common postoperative complications were intra-abdominal infection (9/76, 11.8%), followed by pulmonary infection (5/76, 6.6%) and transfusion (4/76, 5.3%), respectively. While significant difference was found in Grade ≥ III severe postoperative complications in two groups (0 vs. 17.1%, *P* = 0.001). Six (17.1%) patients suffered major complications (grade III–V) requiring invasive interventions in the LG group; three (8.6%) patients with anastomotic leakage recovered after active abodominal drainage; two (5.7%) patients with pulmonary infection recovered after ICU care, and one (2.9%) patient died of respiratory failure. No patient died of postoperative complications in the NACT-LG group. Subgroup analyses were performed to investigate the impact of safety about NACT-LG. It was found that most subgroups of LG group were at great risks of postoperative complications, especially in male, PS = 0, Tumor size ≥ 5 and cT4 groups (Fig. 2).

**Table 3:** Postoperative complications in the NACT-LG and LG groups.

	NACT-LG (n = 41)	LG (n = 35)	P
Grade I	0 (0)	0 (0)	NA
Grade II	9 (22.0)	10 (28.5)	0.680
Transfusion	2 (4.9)	2 (5.7)	
Pulmonary infection	2 (4.9)	3 (8.6)	
Intra-abdominal infection	4 (9.8)	5 (14.2)	
Intra-abdominal hemorrhage	1 (2.4)	0 (0)	
Grade III/IV/V	0 (0)	6 (17.1)	0.001
Anastomotic leakage	0 (0)	3 (8.6)	
Pulmonary infection	0 (0)	2 (5.7)	
Respiratory failure	0 (0)	1 (2.9)	

Fisher’s exact test was used as an alternative to Chi-square test when the number in one of the cells is smaller than 5.

*NACT* neoadjuvant chemotherapy,* LG* laparascopic gastrectomy, *NA* not applicable.

### Pathological outcomes

A comparison of pathologies between the two groups was summarized in Table 4. The number of resected lymph nodes did not differ significantly between the two groups, whereas significantly less metastatic lymph nodes were found in the NACT-LG group than in the LG group (1 vs. 8, P = 0.001). Among all patients, there was a greater proportion of less advanced pT stage (T0–2; 29.3% vs. 0%, P < 0.01), less advanced pN stage (N0; 36.6% vs. 0%, P < 0.01), and less advanced pTNM stage (pTNM 0–II; 51.2% vs. 0%, P < 0.01) in the NACT-LG group than in the LG group. In the NACT-LG group, the pathological response was 65.9%. Over half of the patients (51.2%) obtained tumor downstaging in the NACT-LG group. Four patients (9.8%) were considered pathological complete responders after preoperative chemotherapy.

**Table 4:** Comparison of pathologies between the NACT-LG and LG groups.

	NACT-LG (n = 41)	LG (n = 35)	P
Number of resected lymph nodes	37 (12–91)	32 (12–69)	0.081
Number of metastatic lymph nodes	1 (0–36)	8 (1–27)	0.001
**pT stage**			0.008
0–2	12 (29.3)	0 (0)	
3–4	29 (70.7)	35 (100.0)	
**pN stage**			< 0.01
0	15 (36.6)	0 (0)	
1–3	26 (63.4)	35 (100.0)	
**pTNM stage**			< 0.01
0–II	21 (51.2)	0 (0)	
III	20 (48.8)	35 (100.0)	
**Tumor regression grades**			NA
0	4 (9.8)	NA	
1	13 (31.7)	NA	
2	10 (24.4)	NA	
3	14 (34.1)	NA	
Pathological complete response	4 (9.8)	NA	

Fisher’s exact test was used as an alternative to Chi-square test when the number in one of the cells is smaller than 5.

*NACT* neoadjuvant chemotherapy, *LG* laparascopic gastrectomy, *NA* not applicable.

### Survival

**Figure 3:**
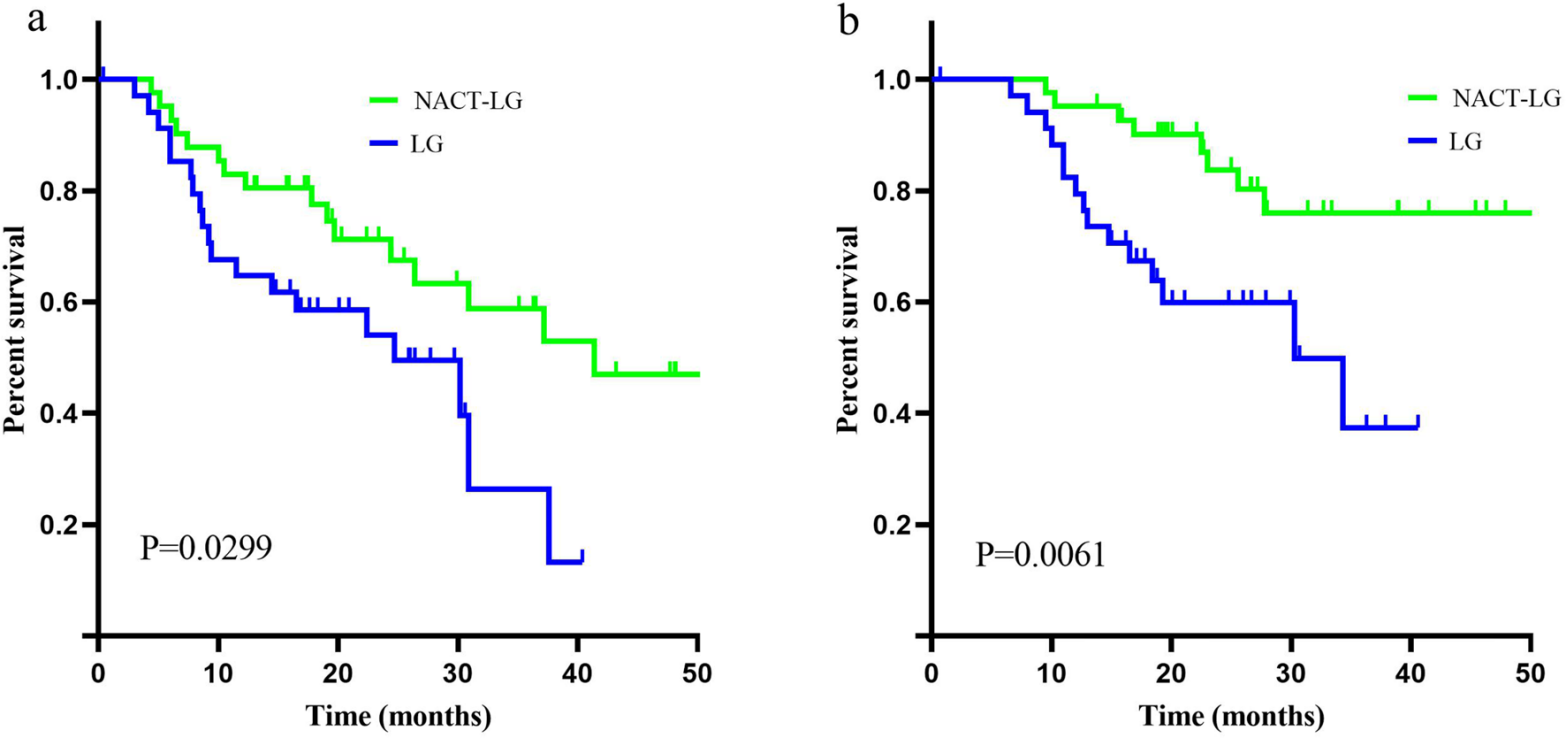
Kaplan–Meier estimates of (**a)** disease-free survival and (**b)** overall survival.

The overall median follow-up was 23.0 months (6.6–71.5) in this study, 25.6 months (9.5–71.5) in the NACT-LG group, and 18.6 months (6.6–40.6) in the LG group, respectively. The survival curves were shown in Fig. 3. The 3-year disease-free survival rates in the NACT-LG group and LG group were 14.4% and 5.7% (P = 0.0299), respectively. The 3-year overall survival rates in the NACT-LG group and LG group were 34.1% and 8.6% (P = 0.0061), respectively (Fig. 3).

## Discussion

LAGC is characterized by low radical resection rate, high relapse rate and high mortality. Laparoscopic surgery combined with perioperative therapy has been widely accepted as the mainstream treatment. However, only few studies focused on the safety and efficacy of laparoscopic surgery in patients after NACT. To our best of knowledge, the present study is the first head-to-head comparison of NACT-LG and LG, and further evaluate the impact of treatment selections on postoperative complications. The present study revealed that NACT-LG increased safety and offered survival benefits in patients with LAGC.

We found that LG following NACT did not increase the severity of surgical trauma, and shorten duration of postoperative hospital stay, which results from less postoperative complications. In addition, operation time was comparable between NACT-LG and LG, and the postoperative complications decreased significantly in NACT-LG group, which was different with a previous study reported by An et al.^8^. It was reported that the negative effects of chemotherapy on operation might be overcome by laparoscope. Laparoscopic surgery offers amplifying visual, better exposure of anatomical hierarchy, and then contributes to delicate anatomy of blood vessels and lymphatic vessels^19^. To further evaluate the impact of NACT-LG regarding postoperative complications, we performed a subgroup analysis and found that patients undergoing NACT-LG were at less risk of developing postoperative complications in most subgroups, especially in male, PS = 0, Tumor size ≥ 5 and cT4 groups. Better compliance and potential benefits to preoperative chemotherapy and reduction of the primary tumor in these patients may be the reasons of less postoperative complications. On the other hand, adverse effects caused by chemotherapy can be attenuated by optimized perioperative care. For instance, malnutrition caused by gastrointestinal side effects can be treated by adequate perioperative nutrition.

Micro-metastases outside the surgical region and microscopic positive margin were the main causes of treatment failure in LAGC^20–22^. NACT may benefit these patients by eradicating metastases and potentially downstaging tumor, thereby increasing the R0 resection rate. Among 41 patients receiving NACT in this study, the pathological response was 65.9%, in concordance with previous studies^23,24^. Over half of patients achieved tumor downstaging, which seemed attributable to the effects of NACT. Moreover, our study demonstrated the benefits of LG following NACT compared with immediate surgery, with an increase of 18 percent in the R0 resection rate (95.1% vs. 77.1%, P = 0.049) and a reduction in the number of metastatic lymph nodes (1 vs. 8, P = 0.001).

The MAGIC trial and the EORTC study disagreed about the survival benefits of perioperative chemotherapy in resectable gastric cancer patients^7,25^. Since proportions of D2 dissection were different among two studies (MAGIC: 42.5%; EORTC: 95.7%), whether patients with D2 gastrectomy benefited from NACT remained unclear. The present study, with all patients received a D2 lymph node dissection, showed that 3-year DFS and OS were superior for NACT-LG than LG (DFS: 14.4% *vs.* 5.7%, OS: 34.1% *vs.* 8.6%). We speculated that sufficient preoperative chemotherapy courses and better compliance to chemotherapy may enhance the positive effects of NACT on survival. However, multi-center, large-sample clinical trials were required to verify this hypothesis in the future.

Despite positive findings mentioned above, there were several limitations in this study. First, as a retrospective study with non-randomized patient groups, selection bias were inevitable in this study. Second, the generalizability of findings drawn from a single-center study may be limited. Third, the follow-up period was too short to analyze the longer survival situation.

## Conclusions

In summary, for patients with LAGC who underwent LG, this study provided supportive evidence favoring the application of NACT. NACT increase the safety of LG for patients with LAGC and offer higher R0 resection rate and better disease-free and overall survival. For patients with LAGC, LG following NACT should be the priority treatment.

## Data Availability

The data that support the findings of this study are available from the corresponding author upon reasonable request.
